# Immunoglobulin (Ig)A seropositivity against SARS-CoV-2 in healthcare workers in Israel, 4 April to 13 July 2020: an observational study

**DOI:** 10.2807/1560-7917.ES.2021.26.48.2001690

**Published:** 2021-12-02

**Authors:** Yaniv Lustig, Carmit Cohen, Asaf Biber, Hanaa Jaber, Yael Becker Ilany, Victoria Indenbaum, Sharon Amit, Michal Mandelboim, Ella Mendelson, Gili Regev-Yochay

**Affiliations:** 1 Central Virology Laboratory, Ministry of Health, Tel-Hashomer, Israel; 2 Sackler Faculty of Medicine, Tel-Aviv University, Israel; 3 Infection Prevention & Control Unit, Sheba Medical Center, Ramat-Gan, Israel; 4 Microbiology Laboratory, Sheba Medical Center, Ramat-Gan, Israel

**Keywords:** COVID-19, SARS-CoV-2, ELISA, serosurveillance, healthcare workers, IgA

## Abstract

**Introduction:**

The COVID-19 pandemic has put healthcare workers (HCW) at significant risk. Presence of antibodies can confirm prior severe acute respiratory syndrome coronavirus 2 (SARS-CoV-2) infection.

**Aim:**

This study investigates the prevalence of IgA and IgG antibodies against SARS-CoV-2 in HCW.

**Methods:**

Performance of IgA and IgG antibody ELISA assays were initially evaluated in positive and negative SARS-CoV-2 serum samples. IgA and IgG antibodies against SARS-CoV-2 were measured in 428 asymptomatic HCW. We assessed the risk of two groups: HCW with high exposure risk outside work (HROW) residing in areas where COVID-19 was endemic (n = 162) and HCW with high exposure risk at work (HRAW) in a COVID-19 intensive care unit (ICU) (n = 97).

**Results:**

Sensitivities of 80% and 81.2% and specificities of 97.2% and 98% were observed for IgA and IgG antibodies, respectively. Of the 428 HCW, three were positive for IgG and 27 for IgA. Only 3/27 (11%) IgA-positive HCW had IgG antibodies compared with 50/62 (81%) in a group of previous SARS-CoV-2-PCR-positive individuals. Consecutive samples from IgA-positive HCW demonstrated IgA persistence 18–83 days in 12/20 samples and IgG seroconversion in 1/20 samples. IgA antibodies were present in 8.6% of HROW and 2% of HRAW.

**Conclusions:**

SARS-CoV-2 exposure may lead to asymptomatic transient IgA response without IgG seroconversion. The significance of these findings needs further study. Out of work exposure is a possible risk of SARS-CoV-2 infection in HCW and infection in HCW can be controlled if adequate protective equipment is implemented.

## Introduction

During December 2019, the severe acute respiratory syndrome coronavirus 2 (SARS-CoV-2), which causes coronavirus disease (COVID-19), was identified in Wuhan, China [1] and since then has spread worldwide [2]. As of 22 Oct 2021, there have been over 242.3 million COVID-19 cases and 4.9 million deaths [3].

Acute COVID-19 is primarily diagnosed by quantitative reverse transcriptase-polymerase chain reaction (qRT-PCR) to detect SARS-CoV-2 RNA [4] and can be used to characterise the incidence of the disease. To assess the prevalence of COVID-19 in the population and prior exposure in individuals, numerous serological kits that measure antibody levels against SARS-CoV-2 have been developed [5]. Because neutralising abilities are derived from IgG antibodies, most serological tests aim at detecting IgG levels. In addition, several recent studies of samples from acute and past COVID-19 cases demonstrated that IgG, IgA and IgM antibody levels are upregulated simultaneously following infection [6,7], suggesting that IgG levels alone may be sufficient for determining past exposure [8]. Interestingly, a recent study comparing IgG and IgM antibodies in asymptomatic and symptomatic qRT-PCR-positive individuals demonstrated that asymptomatic individuals had a weaker immune response to SARS-CoV-2 infection and rapid decline in IgG levels [9], although other studies found that IgG levels against the spike proteins were sustained for 5–7 months after infection [10].

IgA is the major immunoglobulin at the viral point of entry at the mucosal surfaces and is expected to neutralise SARS-CoV-2 before it binds to epithelial cells, but IgA’s role in SARS-CoV-2 infections is not clear [11,12]. Although serum circulating IgA functionally differs from mucosal IgA, the former possesses neutralising abilities and is expected to reflect the latter activity in the upper airway mucosa [13]. A recent study has further highlighted the connection between disease severity and sustainability of IgA high titres [11]. Therefore, evaluation of IgA in serum of asymptomatic individuals or with negative qRT-PCR results may reflect the immune response performance in controlling COVID-19 and will aid in predicting disease outcomes.

Data suggest that a significant part of COVID-19 infection is asymptomatic [14]. Serosurveillance may assist in assessing the effectiveness of protective measures and detecting asymptomatic carriers for control and breech of infection networks [15]. Therefore, it is important to assess the rates of asymptomatic carriers in healthcare workers (HCW) who are facing potential community and hospital exposure.

Here, we studied the seroprevalence of IgA and IgG antibodies against SARS-CoV-2 in asymptomatic HCW with no known history of COVID-19 at the Sheba Medical Center during the early stages of the COVID-19 pandemic.

## Methods

### Setting

The Sheba Medical Center is the largest tertiary medical centre in Israel, with 1,400 acute care beds, 200 rehabilitation beds and 9,342 healthcare workers (HCWs), including 1,855 physicians, 2,847 nurses, 1,992 para-medical staff (physiotherapists, etc.) and 2,648 administrative personnel.

### Study design and population

**Figure 1:**
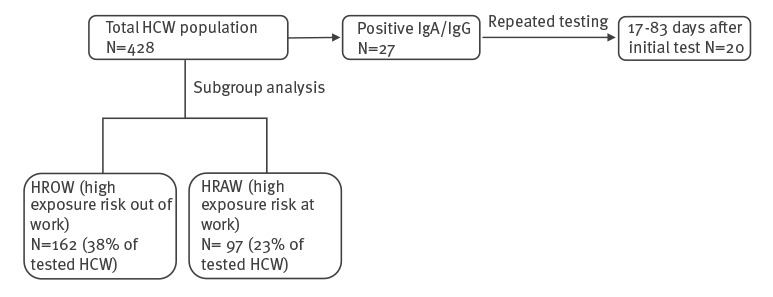
Flowchart of the study design, healthcare worker SARS-CoV-2 seroprevalence study, Ramat Gan, Israel, 4 April 2020–13 July 2020

HCW: healthcare workers; HRAW: high risk at work; HROW: high risk outside work; SARS-CoV-2: severe acute respiratory syndrome coronavirus

Between 4 April and 13 July 2020, we conducted a seroprevalence study of HCW at the Sheba Medical Centre (Figure 1). Participants responding to our call were from medical departments, laboratories, paramedical facilities and service providing departments. HCW who were diagnosed with COVID-19 before the survey were excluded. We sampled volunteers’ blood for serology and obtained nasopharyngeal and oropharyngeal swabs for qRT-PCR. Volunteers’ age, sex, working department, position and home residence were registered. Additionally, the volunteers received a questionnaire containing a list of reported COVID-19 symptoms and were asked to mark any symptoms they experienced in the 2 weeks before the surveillance. HCW presenting with positive immunoglobulin expression were invited for follow-up serology.

We were interested in assessing the risk of two particular groups: (i) HCW with high exposure risk outside work (HROW) residing in areas where rates of identified COVID-19 cases exceeded 21 per 100,000 residents and where the Israeli Ministry of Health declared the areas endemic (red cities) and (ii) HCW with high exposure risk at work (HRAW), i.e., HCW working in the COVID-19 intensive care unit (ICU). During the study period, HCW working in the COVID-19 ICU were instructed to avoid any contacts apart from their household and work. Furthermore, if a household member was ill, they were given a temporary room within the hospital area. The personal protective equipment (PPE) of HCW working in the COVID-19 ICU included a coverall, N95 face mask, face-shield, gloves and shoe leggings. None of the participants belonged to more than one of the two groups (Table 1).

**Table 1:** Characteristics of healthcare workers, healthcare worker SARS-CoV-2 seroprevalence study, Ramat Gan, Israel, 4 April 2020–13 July 2020 (n = 428)

Characteristics	All HCW		HROW group		HRAW group	
	n	%	n	%	n	%
Total population	428	100	162	38	97	23
Sex (female)	287	67	116	71	50	51
Age Mean (range)	40 (18–72)		42 (18–72)		38 (25–69)	
Range of confirmed COVID-19 cases / 100,000 population in place of residence^a^	–		21.1–89.4		0–5.8	
Direct patient contact^b^	282	66	48	30	94	97
SARS-CoV-2 qRT-PCR-positive	2	0.4	2	1.2	0	0
Experiencing symptoms within last 2 weeks	75	17.5	19	12	10	10

COVID-19: coronavirus disease; HCW: healthcare worker; HRAW: high risk at work; HROW: high risk outside work; qRT-PCR: quantitative reverse transcription-polymerase chain reaction; SARS-CoV-2: severe acute respiratory syndrome coronavirus 2.

^a^ As published by the Israeli Ministry of Health on 30 April 2020.

^b^ Practitioners and nurses.

To define the sensitivity and specificity of the test, we assessed IgA and IgG antibody levels in sera obtained from 124 qRT-PCR-positive individuals up to 14 days (< 14, early positive COVID-19) and equal or above 14 days (≥ 14, positive COVID-19) post onset of symptoms (PSO) [4] and from sera obtained before September 2019 from 157 (for IgG) and 181 (for IgA) healthy individuals requesting their polio immunisation status (termed negative COVID-19) (Table 2).

**Table 2:** Evaluation of ELISA assay performance and IgA and IgG seroprevalence, healthcare worker SARS-CoV-2 seroprevalence study, Ramat Gan, Israel, 4 April 2020–13 July 2020 (n = 428)

	Test performance						HCW seroprevalence					
Antibody type	Negative SARS-CoV-2^a^		Early positive COVID-19^b^		Positive COVID-19^c^		All HCW		HROW group		HRAW group	
	Positive /total	Specificity (95% CI)	Positive/total	Sensitivity (95% CI)	Positive /total	Sensitivity (95% CI)	Positive /total	% (95% CI)	Positive/total	% (95% CI)	Positive/total	% (95% CI)
**IgA**	5/181	97.2 (93.3–98.9)	34/69	49.2 (37.1–61.4)	44/55	80 (66.6–89.1)	27/428	6.3 (4.3–9.2)	14/162	8.6 (5.2–14)	2/97	2 (0.6–7.2)
**IgG**	3/157	98 (94–99.5)	19/69	27.5 (17.8–39.8)	45/55	81.8 (68.6–90.4)	3/428	0.7 (0.24–2)	1/162	0.6 (0.1–3)	0/97	0 (0–3.8)

CI: confidence interval; COVID-19: coronavirus disease; HCW: healthcare worker; HRAW: high risk at work; HROW: high risk outside work; qRT-PCR: quantitative reverse transcription-polymerase chain reaction; SARS-CoV-2: severe acute respiratory syndrome coronavirus 2.

^a^ Sera before September 2019.

^b^ Sera from qRT-PCR-positive COVID-19 individuals < 14 days.

^c^ Sera from recovered COVID-19 individuals ≥ 14 days.

### PCR testing

For qRT-PCR, nasopharyngeal and oropharyngeal swabs were placed in 3 mL of universal transport medium (UTM) or viral transport medium (VTM). Tests were performed according to manufacturers’ instructions on various platforms: Allplex 2019-nCoV (Seegene, South Korea), NeuMoDx SARS-CoV-2 assay (NeuMoDx Molecular, Ann Arbor, Michigan, US), and Xpert, Xpress SARS-CoV-2 (Cepheid, Sunnyvale, CA, US).

### IgA and IgG antibody analysis

IgA and IgG antibodies against the S1 domain of the spike protein of SARS-CoV-2 (expressed recombinantly in the human cell line HEK 293) were detected by a semiquantitative enzyme-linked immunoassay (ELISA) (anti-SARS-CoV-2 ELISA IgG and anti-SARS-CoV-2 ELISA IgA, Euroimmun, Lübeck, Germany). Samples were tested according to the manufacturer’s instructions. ELISA index value was defined as the ratio between sample and cut-off optical densities (OD). An ELISA index value below 0.9 was considered negative, between 0.9 and 1.1 was considered borderline and equal or above 1.1 was considered positive. Borderline results were considered negative.

### Statistical analysis

Scatter plot and correlation analyses were performed using GraphPad Prism 9.0 (GraphPad Software, Inc., San Diego, CA, US) by two-tailed parametric t-test means with confidence intervals (CI) of 95%. We compared the rates of seropositivity in the two high risk populations to the control negative group, from whom the specificity of the ELISA test was calculated using Fisher’s exact test and chi-squared test.

### Ethical statement

The institutional review board of Sheba Medical Center approved the study and waived the requirement for informed consent on the basis of voluntary participation and preserving participants’ anonymity.

## Results

### IgA and IgG ELISA performance

**Figure 2:**
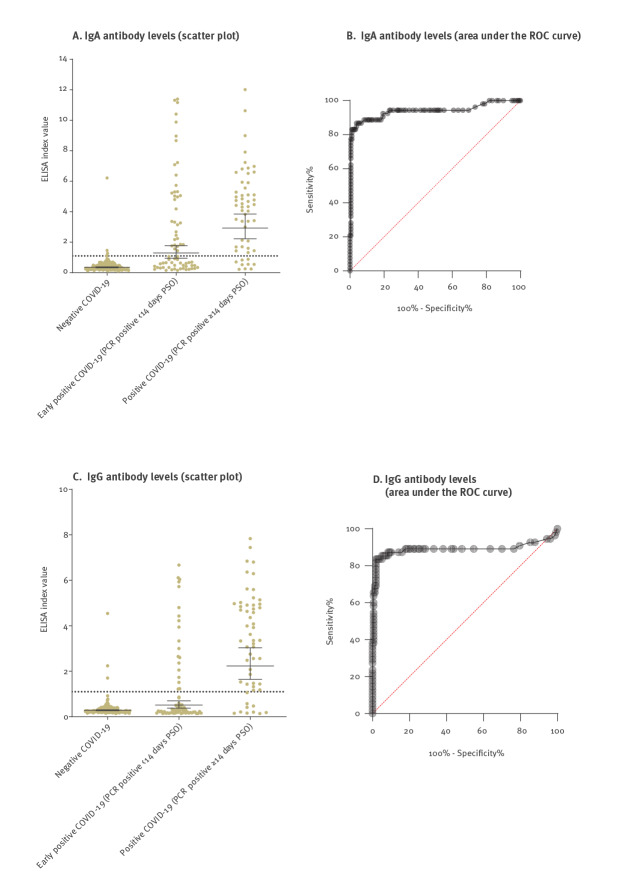
Performance of IgA and IgG ELISA, healthcare worker SARS-CoV-2 seroprevalence study, Ramat Gan, Israel, 4 April 2020–11 May 2020

COVID-19: coronavirus disease; ELISA: enzyme-linked immunoassay; PCR: polymerase chain reaction; ROC: receiver operating characteristic; SARS-CoV-2: severe acute respiratory syndrome coronavirus 2.

We first evaluated the performance of the ELISA. IgG and IgA sensitivity reached 27.5% (95% CI: 17.8–39.8) and 49.2% (95% CI: 37.1–61.4) in the first 2 weeks PSO and increased to 81.2% (95% CI: 68.6–90.4) and 80% (95% CI: 66.6–89.1), respectively, 2 weeks PSO. Specificity was 98% for IgG and 97.2% for IgA. Overall, the area under the ROC curve was 0.89 and 0.91 for IgA and IgG, respectively (Figure 2 and Table 2).

Scatter plots: Negative COVID-19 (Non-COVID) individuals; SARS-CoV-2-positive COVID-19 persons less than 14 days post symptom onset (PCR positive < 14 days PSO); and SARS-CoV-2-positive COVID-19 persons more than or equal to 14 days post symptom onset (PCR positive ≥ 14 days PSO). Area under the ROC curve cut-off level is presented as a dashed line. Geometric mean titre and +/− 95% confidence interval are shown.

### IgA and IgG SARS-CoV-2 seroprevalence in healthcare workers

Of the 428 HCW included in this study (mean age 40 and 287 (67%) women), 75 (17.5%) reported experiencing symptoms in the 2 weeks before sample collection, and two were positive for SARS-CoV-2 RNA (one was positive for both IgG and IgA and one for only IgA antibodies against SARS-COV-2) at the time of recruitment. The two positive participants did not report any symptoms (Table 1). Of the 428 HCW, three were positive for both IgG and IgA (95% CI: 0.24–2) and 27 were positive for only IgA (95% CI: 4.3–9.2) (Table 2). None of the IgA and IgG-positive individuals reported any previous symptoms. Interestingly, there was a substantial difference in the proportion of IgA- and IgG-positive individuals between SARS-CoV-2 qRT-PCR-positive persons and the HCW. Overall, 50/62 (81%) of SARS-CoV-2 qRT-PCR-positive persons had both IgA and IgG antibodies with an IgA mean ELISA index value of 4.4. However, only 3/27 HCW with a positive IgA had detectable IgG and their IgA mean ELISA index value was 2.

To examine antibody kinetics, all HCW with positive IgA were asked to have a repeated test at least 14 days after the initial result (Figure 1). Of the 27 IgA positive HCW, 20 returned for a second test 17 to 83 (mean of 60.1 days) days after the first test; 12 of the samples were persistently IgA positive and two were persistently IgG positive (Table 3). In addition, one sample seroconverted to IgG with simultaneous decrease in IgA, and seven samples demonstrated an IgA decrease, either to a borderline value (four samples) or a negative value (three samples).

**Table 3:** Antibody kinetics of IgA-positive participants, healthcare worker SARS-CoV-2 seroprevalence study, Ramat Gan, Israel, 4 April 2020–13 July 2020 (n = 20)

Participant number	First sample		Second sample		Time (days) between samples
	IgA result (ratio)	IgG result (ratio)	IgA result (ratio)	IgG result (ratio)	
**1**	Pos (1.17)	Neg	Pos (2.00)	Neg	83
**2**	Pos (1.55)	Neg	Pos (2.39)	Neg	80
**3**	Pos (2.61)	Neg	Pos (2.40)	Neg	79
**4**	Pos (2.22)	Neg	Neg	Neg	79
**5**	Pos (1.17)	Neg	Border (1.05)	Neg	79
**6**	Pos (1.17)	Neg	Border (0.80)	Neg	79
**7**	Pos (1.24)	Neg	Border (0.82)	Neg	78
**8**	Pos (2.3)	Neg	Pos (2.76)	Neg	73
**9**	Pos (2.18)	Neg	Neg	Neg	73
**10**	Pos (1.67)	Neg	Pos (1.65)	Neg	72
**11**	Pos (1.16)	Neg	Pos (1.35)	Neg	72
**12**	Pos (1.29)	Neg	Pos (1.83)	Neg	69
**13**	Pos (1.30)	Neg	Pos (1.28)	Neg	67
**14**	Pos (1.47)	Neg	Pos (2.47)	Neg	52
**15**	Pos (1.90)	Neg	Neg	Neg	36
**16**	Pos (1.64)	Neg	Border (1.06)	Pos (1.24)	24
**17**	Pos (7.97)	Pos (1.54)	Pos (6.64)	Pos (2.53)	22
**18**	Pos (1.40)	Border (0.96)	Pos (1.53)	Neg	19
**19**	Pos (6.67)	Pos (6.42)	Pos (6.09)	Pos (13.23)	18
**20**	Pos (1.14)	Border (1.00)	Border (0.96)	Border (0.8)	17

Neg: negative; Pos: positive; SARS-CoV-2: severe acute respiratory syndrome coronavirus 2.

### Comparison of two groups of healthcare workers

Among the 428 HCW, 162 residing in COVID-19 endemic areas were considered a high risk outside work group (HROW), and 97 HCW working in the COVID-19 ICU were considered a high risk at work group (HRAW). The mean age of the participants was 42 and 38, respectively. In the HROW group, 48 HCW were practitioners and nurses who had direct contact with patients; in the HRAW group, 94 HCW were practitioners and nurses who had direct contact with patients (Table 1). IgG antibodies were detected in serum from one HROW HCW (95% CI: 0.1–3) and none of the HRAW participants (95% CI: 0–3.8), and IgA antibodies were detected in 14 HROW participants (95% CI: 5.2–14) and two HRAW participants (95% CI: 0.6–7.2) (Table 2). Although IgG levels were not significantly higher than the negative control group, which was assessed to define specificity (i.e., they were not higher than the false positivity rate), IgA levels for HCW with HROW but not HRAW were significantly higher than the false positive rate of the non-COVID group (p = 0.01).

## Discussion

The COVID-19 pandemic has resulted in high exposure, high infection and high isolation rates among frontline HCW [16]. Thus, identifying HCW who were exposed to SARS-CoV-2 and in whom infections remained undetected and particularly identifying those with SARS-CoV-2 antibodies who are presumably immune to COVID-19 may be of high importance.

Our study found a 0.7% IgG seroprevalence for our study population of 428 HCW (3/428) at the early stage of the COVID-19 pandemic in Israel. This result agrees with the Ministry of Health assessments of between 0.5% and 1% IgG seroprevalence in Israel and only about 16,000 qRT-PCR-positive COVID-19 cases at the time the study, were confirmed. At least in terms of IgG antibodies, this result suggests that Sheba HCW were not exposed more than the general Israeli population.

One of the most important findings of this study is that IgA seroprevalence is about 6% among asymptomatic HCW with unknown previous exposure or detection of SARS-CoV-2. During the COVID-19 pandemic, numerous serological kits have been developed and validated [17]; however, most of these assays are aimed at detecting IgG antibodies as these are believed to be sufficient to confer protection against SARS-CoV-2 infection both at the individual and population level [18]. Importantly, to date only one commercial company has developed an IgA assay, which was used in this study and which has been evaluated by several other studies [19,20]. The performance of this and other serological assays varies between studies, an observation that may stem from different cohorts or immunological backgrounds. Also, limited cross reactivity of the IgA assay with seropositive hepatitis C virus samples was recently found [21], suggesting that caution should be exercised when interpreting our results. Therefore, to increase the specificity of the IgA assay, we used both non-COVID-19 and SARS-CoV-2 qRT-PCR-positive individuals as controls and considered IgA borderline results to be negative. Our validation with samples obtained from both non-COVID-19 and SARS-CoV-2 qRT-PCR-positive persons from Israel showed that the seropositivity results from HCW residing in highly endemic areas (i.e., with high exposure risk outside work) are significantly higher than in negative controls and higher than in HCW who are well protected by PPE, even if working in departments with potential high exposure risk.

Most interesting are the differences in antibody profiles we identified between SARS-CoV-2 qRT-PCR-positive and our cohort of HCW. Unlike individuals diagnosed with COVID-19, most HCW exposed to SARS-CoV-2 were serologically positive only for IgA antibodies with a comparably smaller mean ELISA index value. Indeed, a study evaluating the seroprevalence of healthcare professionals in Germany also found specific IgA but not IgG seropositivity in 19/217 participants and only three healthcare professionals had both IgA and IgG [22]. In our study, despite an IgA response that was sustained for at least several weeks, no IgG seroconversion was observed over time in most cases. Assessment of IgA kinetics overtime in large studies reveals that sustained detection of individuals diagnosed with COVID-19 may predict disease outcomes, and persistence may last for over 3 months [11,23]. Future population-based studies and predictive models should consider such factors to generalise results. Furthermore, it would be interesting to study what types of exposures lead to an exclusive IgA response, or to both IgA and IgG responses in asymptomatic individuals. The implications of these antibody responses should be explored further.

Two intriguing questions originating from this study concern the function of IgA and the lack of IgG antibodies in the participating HCW. It is well documented that IgA antibodies have neutralising activity [24,25] and recently IgA was demonstrated to be a potent SARS-CoV-2 neutralising agent [26]. Our results suggest that, unlike SARS-CoV-2-positive individuals, in most IgA-positive HCW with a negative qRT-PCR result for SARS-CoV-2, IgG does not develop, although IgA antibodies have lower titres and can be sustained for at least several weeks. Since IgA was shown to dominate the early neutralising antibody response to SARS-CoV-2 [26], it is possible that the development of IgA antibodies can be sufficient to overcome SARS-CoV-2 infection under certain circumstances such as low level or asymptomatic infection. Under these conditions, SARS-CoV-2 infection may be cleared before IgG antibodies can be produced. Despite being the most abundant antibody isotype present at mucosal surfaces and the second most abundant in serum [27], IgA levels need more evaluation to be useful for diagnostics. It will be interesting to examine the role of IgA antibodies in other viral infections and to explore the differences between serum and secretory IgA activities.

Our study also answers one key concern: the effectiveness of PPE worn by HCW working long hours with COVID-19 patients. To assess the risk of exposure at work and the risk of exposure at home, we examined the prevalence of antibodies against COVID-19 in two groups of HCW: one group of HCW working 8-hour shifts and returning to their residence in COVID-19 endemic areas and one group of HCW working in the COVID-19 ICU under restricted residential conditions. Interestingly, our results identified increased IgA but not IgG seroprevalence in the HCW with HROW, but no significant increase was detected in the HRAW group. This suggests that HCW working in the COVID-19 ICU were more protected from COVID-19 than other HCW at the Sheba Medical Center. This protection is probably due to the strict PPE policy and the isolation of these ICU personnel from their families and friends.

Limitations of this study include the relatively small number of samples used for assessing the seroprevalence of both high risk at home and at work groups and the substantial proportion of participants reporting symptoms, which might not be proportional to the general population. Therefore, we were not able to perform in-depth analysis and assess correlates for antibody seropositivity. Larger studies of HCW that include departments with differing exposure risks are urgently needed to unravel the impact of the COVID-19 pandemic on HCW from different demographic characteristics and work conditions.

In conclusion, this study, which was conducted during the early stages of the COVID-19 pandemic in Israel, corroborates the importance of adequate PPE for HCW protection against SARS-2-CoV infection and highlights a specific IgA antibody seropositivity among asymptomatic HCW.

## References

[1] Wu F Zhao S Yu B Chen YM Wang W Song ZG A new coronavirus associated with human respiratory disease in China. Nature. 2020;579(7798):265-9. 10.1038/s41586-020-2008-3 32015508 PMC7094943

[2] Rafiq D Batool A Bazaz MA . Three months of COVID-19: A systematic review and meta-analysis. Rev Med Virol. 2020;30(4):e2113. 10.1002/rmv.2113 32420674 PMC7267122

[3] Singh S, McNab C, Olson RM, Bristol N, Nolan C, Bergstrøm E, et al. How an outbreak became a pandemic: a chronological analysis of crucial junctures and international obligations in the early months of the COVID-19 pandemic. Lancet. 2021;8:S0140-6736(21)01897-3. 10.1016/S0140-6736(21)01897-3. 34762857 PMC8575464

[4] Corman VM Landt O Kaiser M Molenkamp R Meijer A Chu DKW Detection of 2019 novel coronavirus (2019-nCoV) by real-time RT-PCR. Euro Surveill. 2020;25(3). 10.2807/1560-7917.ES.2020.25.3.2000045 31992387 PMC6988269

[5] Özçürümez MK Ambrosch A Frey O Haselmann V Holdenrieder S Kiehntopf M SARS-CoV-2 antibody testing-questions to be asked. J Allergy Clin Immunol. 2020;146(1):35-43. 10.1016/j.jaci.2020.05.020 32479758 PMC7256507

[6] Long QX Liu BZ Deng HJ Wu GC Deng K Chen YK Antibody responses to SARS-CoV-2 in patients with COVID-19. Nat Med. 2020;26(6):845-8. 10.1038/s41591-020-0897-1 32350462

[7] Wang Y Zhang L Sang L Ye F Ruan S Zhong B Kinetics of viral load and antibody response in relation to COVID-19 severity. J Clin Invest. 2020;130(10):5235-44. 10.1172/JCI138759 32634129 PMC7524490

[8] Indenbaum V Koren R Katz-Likvornik S Yitzchaki M Halpern O Regev-Yochay G Testing IgG antibodies against the RBD of SARS-CoV-2 is sufficient and necessary for COVID-19 diagnosis. PLoS One. 2020;15(11):e0241164. 10.1371/journal.pone.0241164 33227020 PMC7682882

[9] Long QX Tang XJ Shi QL Li Q Deng HJ Yuan J Clinical and immunological assessment of asymptomatic SARS-CoV-2 infections. Nat Med. 2020;26(8):1200-4. 10.1038/s41591-020-0965-6 32555424

[10] Ripperger TJ, Uhrlaub JL, Watanabe M, Wong R, Castaneda Y, Pizzato HA, et al. Orthogonal SARS-CoV-2 Serological Assays Enable Surveillance of Low-Prevalence Communities and Reveal Durable Humoral Immunity. Immunity. 2020;53(5):925-33 e4. 10.1016/j.immuni.2020.10.004 PMC7554472 33129373

[11] Tang J Ravichandran S Lee Y Grubbs G Coyle EM Klenow L Antibody affinity maturation and plasma IgA associate with clinical outcome in hospitalized COVID-19 patients. Nat Commun. 2021;12(1):1221. 10.1038/s41467-021-21463-2 33619281 PMC7900119

[12] Chao YX Rötzschke O Tan EK . The role of IgA in COVID-19. Brain Behav Immun. 2020;87:182-3. 10.1016/j.bbi.2020.05.057 32454136 PMC7245198

[13] Russell MW Moldoveanu Z Ogra PL Mestecky J . Mucosal Immunity in COVID-19: A Neglected but Critical Aspect of SARS-CoV-2 Infection. Front Immunol. 2020;11:611337. 10.3389/fimmu.2020.611337 33329607 PMC7733922

[14] Huff HV Singh A . Asymptomatic transmission during the COVID-19 pandemic and implications for public health strategies. Clin Infect Dis. 2020. 10.1093/cid/ciaa654 PMC7314132 32463076

[15] Peeling RW Wedderburn CJ Garcia PJ Boeras D Fongwen N Nkengasong J Serology testing in the COVID-19 pandemic response. Lancet Infect Dis. 2020;20(9):e245-9. 10.1016/S1473-3099(20)30517-X 32687805 PMC7367660

[16] Chou R Dana T Buckley DI Selph S Fu R Totten AM . Epidemiology of and Risk Factors for Coronavirus Infection in Health Care Workers: A Living Rapid Review. Ann Intern Med. 2020;173(2):120-36. 10.7326/M20-1632 32369541 PMC7240841

[17] Oved K, Olmer L, Shemer-Avni Y, Wolf T, Supino-Rosin L, Prajgrod G, et al. Multi-center nationwide comparison of seven serology assays reveals a SARS-CoV-2 non-responding seronegative subpopulation. EClinicalMedicine. 2020;29-30:100651. 10.1016/j.eclinm.2020.100651 PMC7676374 33235985

[18] Huang AT Garcia-Carreras B Hitchings MDT Yang B Katzelnick LC Rattigan SM A systematic review of antibody mediated immunity to coronaviruses: kinetics, correlates of protection, and association with severity. Nat Commun. 2020;11(1):4704. 10.1038/s41467-020-18450-4 32943637 PMC7499300

[19] Charlton CL Kanji JN Johal K Bailey A Plitt SS MacDonald C Evaluation of six commercial mid to high volume antibody and six point of care lateral flow assays for detection of SARS-CoV-2 antibodies. J Clin Microbiol. 2020;58(10):e01361-20. 10.1128/JCM.01361-20 32665420 PMC7512179

[20] Montesinos I Gruson D Kabamba B Dahma H Van den Wijngaert S Reza S Evaluation of two automated and three rapid lateral flow immunoassays for the detection of anti-SARS-CoV-2 antibodies. J Clin Virol. 2020;128:104413. 10.1016/j.jcv.2020.104413 32403010 PMC7198434

[21] Needle R Gilbert L Zahariadis G Yu Y Dalton-Kenny H Russell RS Serological Evaluation of Human Antibodies of the Immunoglobulin Class A and G Against SARS-CoV-2 in Serum Collected in Newfoundland and Labrador. Viral Immunol. 2021;34(3):182-9. 10.1089/vim.2020.0199 33739895

[22] Behrens GMN Cossmann A Stankov MV Witte T Ernst D Happle C Perceived versus proven SARS-CoV-2-specific immune responses in health-care professionals. Infection. 2020;48(4):631-4. 10.1007/s15010-020-01461-0 32524515 PMC7286418

[23] Gudbjartsson DF Norddahl GL Melsted P Gunnarsdottir K Holm H Eythorsson E Humoral Immune Response to SARS-CoV-2 in Iceland. N Engl J Med. 2020;383(18):1724-34. 10.1056/NEJMoa2026116 32871063 PMC7494247

[24] de Sousa-Pereira P Woof JM . IgA: Structure, Function, and Developability. Antibodies (Basel). 2019;8(4):E57. 10.3390/antib8040057 31817406 PMC6963396

[25] Hansen IS Baeten DLP den Dunnen J . The inflammatory function of human IgA. Cell Mol Life Sci. 2019;76(6):1041-55. 10.1007/s00018-018-2976-8 30498997 PMC6513800

[26] Huang AT Garcia-Carreras B Hitchings MDT Yang B Katzelnick LC Rattigan SM A systematic review of antibody mediated immunity to coronaviruses: kinetics, correlates of protection, and association with severity. Nat Commun. 2020;11(1):4704. 10.1038/s41467-020-18450-4 32943637 PMC7499300

[27] Davis SK Selva KJ Kent SJ Chung AW . Serum IgA Fc effector functions in infectious disease and cancer. Immunol Cell Biol. 2020;98(4):276-86. 10.1111/imcb.12306 31785006 PMC7217208

